# Optimized precooling combined with SO_2_‐released paper treatment improves the storability of longan (*Dimocarpus longan* Lour.) fruits stored at room temperature

**DOI:** 10.1002/fsn3.1577

**Published:** 2020-04-16

**Authors:** Dongmei Han, Tao Luo, Lu Zhang, Jiaqi Wu, Huitao Wu, Zhenxian Wu, Jianguang Li, Jing Wang, Xuewen Pan

**Affiliations:** ^1^ Institute of Fruit Tree Research Guangdong Academy of Agricultural Sciences/Key Laboratory of South Subtropical Fruit Biology and Genetic Resource Utilization Ministry of Agriculture/Guangdong Province Key Laboratory of Tropical and Subtropical Fruit Tree Research Guangzhou China; ^2^ College of Horticulture South China Agricultural University/Guangdong Provincial Key Laboratory of Postharvest Science of Fruits and Vegetables/Engineering Research Center for Postharvest Technology of Horticultural Crops in South China Ministry of Education Guangzhou China; ^3^ Guangdong Litchi Engineering Research Center/Key Laboratory of Biology and Genetic Improvement of Horticultural Crops (South China) of Ministry of Agriculture Guangzhou China

**Keywords:** Longan (*Dimocarpus longan* Lour.) fruit, orthogonal test, postharvest performance, pre‐cooling, SO_2_‐released paper

## Abstract

Precooling and sulfur dioxide fumigation were proved as effective methods for the preservation of longan (*Dimocarpus longan* Lour.) fruits. However, inadequate precooling and sulfur dioxide fumigation resulted in unexpected losses and short shelf life. A L_9_(3^4^) orthogonal test was conducted to screen out ideal dosage of sodium metabisulfite (factor A), precooling method (factor B), and precooling duration (factor C) to improve the storability of longan fruit stored for 48 hr at room temperature (RT) (25℃). The overall qualities of all of the treated longan fruits after a 48‐hr storage (OQST) and during the 5‐day shelf at 25℃ (OQSF) were better than those of the control fruits. The treated fruits showed brighter fresh color (higher L*, b*, C*, and h° values but lower a* value), higher flavonoid, and chlorophyll contents. Moreover, the SO_2_ residue was concentrated in pericarp but little in aril for any of the 12 treatments. The multivariate variance analysis showed that factor A was dominant to determine both of the OQST and OQSF, while factor B affected the OQST, and factor C affected the OQSF. In total, “0.22% sodium metabisulfite + 4 hr precooling + uncovered precooling” was considered to be an ideal treatment. These results would contribute to improving longan postharvest preservation technology.

## Graphical abstract

The overall qualities of all of the treated longan fruits after a 48‐hr storage (OQST) and during the 5‐d shelf at 25℃ (OQSF) were better than those of the control fruits. The treated fruits showed brighter fresh color (higher L*, b*, C*, and h° values but lower a* value), higher flavonoid, and chlorophyll contents. Factor A (dosage of sodium metabisulfite) was the dominant factor, and “0.22% sodium metabisulfite + 4 hr precooling + uncovered precooling” was considered to be an ideal treatment.

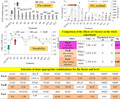

## 1 INTRODUCTION

Longan (*Dimocarpus longan* Lour.), one of the typical tropical and subtropical fruits, is extremely unresistant to a long‐term postharvest storage and transportation. The biggest problem during postharvest for longan is pericarp browning and aril breakdown (Han et al., 2014). The harvested longan fruit might be able to extend its life about 15–30 days when stored at suitable low temperature but only 3–5 days at ambient temperature (ABT) (nearly 32–35℃). The typical deterioration of longan fruits was usually featured with a serious pericarp browning or aril juicy (Han et al., 2015; Luo, Li, et al., 2019). In China's domestic markets, longan fruit is usually stored and transported at ABT, sealed in a foam box with ice bags to keep it cool. Obviously, unfavorable internal environmental conditions, such as higher temperature and more condensate water in the package, resulted in serious postharvest losses. Therefore, it is essential to develop appropriate and effective technologies serviced for ambient temperature logistic and storage of longan fruits.

Precooling not only rapidly reduces the temperature of horticulture products, but also helps the products quickly slowdown its metabolic activity, enter a low energy consumption state and prolongs its shelf life (Brosnan & Sun, 2001; Ladaniya, 2008). As it was known, the precooling method is very important for the efficiency of product cooling. A previous work reported that a forced air precooling combined with suitable packaging can quickly reduce the internal temperature of citrus fruits and shorten the precooling duration (Defraeye et al., 2014). It was worthy to note that the cold‐sensitive fruit was suggested to be gradually cooled for avoiding cold injury (Defraeye, Verboven, Opara, Nicolai, & Cronjé, 2015). More importantly, the precooling practice should be promptly implemented after harvest. A 6‐hr delay of precooling at 30°C resulted in increased water loss rate of strawberry by 50%, decreased hardness by 14%–22%, and significantly declined quality Reichel (Nunes, Brecht, Morais, & Sargent, 1995).

In harvesting season, the respiration rate of longan fruit is very high due to the high orchard temperature (usually 35℃ or higher) and strong sun exposure. Therefore, a precooling practice is necessary to be performed on the longan fruit before its storage. However, longan fruits are usually packaged in cartons or baskets after being air‐cooled in a shade and covered with some leaves to keep moisturizing, which does not achieve the purpose of extending the shelf life. In addition, the conventional and direct precooling methods usually resulted in a high water loss from pericarp of longan fruits without covering. Precooling using ice‐water mixture is also found to be not suitable for longan, which probably aggravates the pericarp browning and rottenness (Lin, Chen, Kong, & Xi, 2007). Previous results indicated that packaging of longan fruits in the films with stronger water permeability leads to more serious pericarp browning (Han et al., 2017). Therefore, the existing precooling methods need to be optimized depending on different storage and transportation purposes.

SO_2_ and its donors (such as sodium metabisulfite) have been widely used for long‐term storage of fresh or processed agricultural products due to its remarkable bleaching action on organic pigments, strong capacity to inhibit bacterial, and act as an antioxidant and inhibitor of enzyme‐catalyzed or nonenzymatic oxidative discoloration and of browning during storage (Li, Yang, Chen, Li, & Liu, 2015; Prabhakar & Mallika, 2014; Wang, Cui, & Fang, 2007). Therefore, it has been widely applied in the processing of food to protect or improve the apparent color and prevent rottenness (Chen, 2010; Hu et al., 2017). Its excellent preservation effect was observed on Chinese herbal medicines (Ji, Li, Peng, & Dong, 2015; Wang et al., 2015), the exported fresh litchi fruits (Kumar, Teotia, Prasad, Varma, & Kumar, 2017), and the table grapes (Carter, Chapman, Gabler, & Brandl, 2015; Liu et al., 2015) during a long‐term storage. SO_2_ fumigation was proved as a key technology for postharvest treatment of international traded fresh longan fruits (Chen, 2013; He et al., 2006). After the fumigation, SO_2_ gas can fully penetrate into the skin and flesh tissue of longan fruit, rapidly bleach the skin by reducing the brown pigments (Pang, Zhang, Gong, & Zhang, 2007; Wu, Han, Ji, & Chen, 1999), reduce the respiration rate, and significantly extend its storage life (Han, Wu, Ji, & Han, 1999). However, the SO_2_ fumigation technology requires particular facilities and fixed sites, as well as a large investment, which inhibited its widely application in domestic market.

In view of its excellent effect on color protection, SO_2_ gas combined with the appropriate precooling was expected to reduce the condensate water in the package, the mildew, and rot of fruit. Based on this thesis, our self‐made SO_2_‐released paper capable of releasing SO_2_ gas (patent application no. 201610227848.7 in China, based on sodium metabisulfite as the main ingredient) has been applied in longan fruit storage experiment. Our previous trial (“SO_2_‐released paper + 5°C precooling”) was applied on longan fruits, which were packed in foam box with ice bags for 44 hr plus 3‐day shelf at 25℃. This treatment significantly reduced the condensate water in package, resulted in a higher shelf marketability rate (up to 91.92%) when compared to the control fruits (up to 80.69%). However, the precooling method and the duration of precooling still need to be investigated and optimized. A L_9_(3^4^) orthogonal test was conducted to investigate the effects of three factors, including the dosage of compound preservative (factor A), precooling method (factor B), and precooling duration (factor C) on the overall qualities of longan fruits after a 48‐hr storage (OQST) within foam boxes and during the shelf (OQSF) at 25℃ and screen out an ideal treatment. The results from this work were expected to help improve postharvest technology of longan fruits and the similar researches on other fruits and vegetables.

## 2 MATERIALS AND METHODS

### 2.1 Fruits, SO_2_‐released paper, and reagents

Longan fruits cv. “Chuliang” with 85%–95% maturity degree were harvested on 26 July 2017 from Huaxiang Orchard in Yangxi, Guangdong Province, China. The environmental temperature of the orchard was 36–37℃. The fruits were harvested with long branches, which were cut short to be 15–20 cm. Fruits with uniformed size and appearance were selected and immediately transported to the laboratory within 3 hr in an air‐conditioned car (about 23℃).

### 2.2 Chemicals

Food‐grade sodium metabisulfite for making SO_2_‐released paper was obtained from Shandong Weifang Lujiu Chemical co., Ltd. The prochloraz and iprodione were, respectively, supplied by Jiangsu Huifeng Agrochemical Co., Ltd. and Jiangsu Kuaida Agrochemical Co., Ltd. Ethanol, acetone, methanol, zirconium oxychloride (ZrOCl_2_.8H_2_O), sodium chloride, mercuric chloride, zinc sulfate, borax, and para‐rose aniline hydrochloride were all analytical reagents and supplied by Sinopharm Chemical Reagent Co., Ltd.

### 2.3 Sterilization

Fruits with no disease and no damage were selected and dipped into solution containing 500 mg/L prochloraz and 1,000 mg/L iprodione for 2 min. The fruits were then taken out and dried at an air conditional room about 25℃.

### 2.4 Package, precooling, storage, and sampling

Sterilized fruits of 2.5 kg were put into a foam box (inner size 36 cm × 26 cm × 10 cm) with a 0.02‐mm thickness polyethylene (PE) bag liner. The foam boxes without sealing were then moved into a refrigeration room. The fruits with or without coverage (see Table 1, factor B) were precooled at 5 ± 0.5°C for 0, 4, 12, or 20 hr (see Table 1, factor C).

**Table 1:** Orthogonal experiment design (three factors and four levels)

Levels	Factors		
	A: sodium metabisulfite (%)	B: precooling method	C: precooling duration (h)
1	0.22	UP	4
2	0.18	PP	12
3	0.14	WP	20
4	0.12	–	–

Abbreviations: PP, precooling with paper, covered with a single layer of newsprint; UP, Uncovered precooling, with no covering; WP, wet precooling, covered with a piece of wet cloth after squeezing.

After the precooling, one bag of 800 g ice was placed under the liner (upon the bottom of the box). A sheet of SO_2_‐released paper containing 0.12%, 0.14%, 0.18%, or 0.22% (w/w) sodium metabisulfite (see Table 1, factor A) was laid upon the top of fruits with the SO_2_ releasing side faced down. After the lid was covered, the foam boxes were sealed with tape and stored in a storage room with constant temperature at 25 ± 1°C for 48 hr. After the room temperature storage, the foam boxes were opened. Thirty fruits from each treatment were sampled: Half of the pericarp and half of the aril were separated from each fruit and immediately frozen in liquid nitrogen, ground, wrapped in a piece of aluminum foil paper, and stored at 80°C until used. Three replicates were performed for each treatment.

### 2.5 Shelf experiment and sampling

After the foam boxes were opened, the fruits with long branches from each treatment were trimmed into single fruit with 3‐ to 5‐cm branch. The trimmed fruits were placed into a transparent plastic box with two holes (diameter 8 mm) in each face. Each box of fruits was weighted about 500 g and stored on the shelf at 25 ± 1°C. Three replicates were performed for each treatment. After the 4‐day shelf experiment, 30 fruits from each treatment were sampled as described above.

### 2.6 Orthogonal test design

A L_9_ (3^4^) orthogonal test of three factors including factor A (dosage of sodium metabisulfite), factor B (different coverage methods during precooling), and factor C (precooling duration) was designed (Table 1).

Twelve treatments designed from the combinations of factors and levels were, respectively, labeled as S1–S12 (Table 2), S9–S12 were derived from the additional level 4 of factor A, and only one repeat for each treatment. The four levels of factor A were set according to the effective dosages of sodium metabisulfite to control decay and result in a SO_2_ residue in longan aril below the national standard limit (30 mg/kg) (Yang et al., 2007, NY 1440–2007, maximum limit of sulfur dioxide residue in tropical fruits in China). The levels of factor B were set according to their gradient moisturizing ability. The levels of factor C were considered according to the fact that the fruit inner temperature might be closed to 5℃ after being precooled for about 12 hr at 5℃. In addition, the fruits with no SO_2_‐released paper and no precooling treatment were marked as control (CK) and repeated three times. The cooled fruits were packed in batches according to the planned precooling duration of different treatments. All of the treatments and CK were unpacked after stored for 48 hr at 25℃.

**Table 2:** Orthogonal array of the experiments on longan fruit stored at 25°C

Treatment	Factors & levels			
	A	B	C	Error
S1	1	1	1	1
S2	1	2	2	2
S3	1	3	3	3
S4	2	1	2	3
S5	2	2	3	1
S6	2	3	1	2
S7	3	1	3	2
S8	3	2	1	3
S9	3	3	2	1
S10	4	1	3	2
S11	4	2	1	3
S12	4	3	2	1
CK	−	−	−	−

### 2.7 Indicators related to commodity

After a 48‐hr storage within foam box at 25℃, no obvious pericarp browning and mildew were observed on the pericarp of the treated and control longan fruits. The differences among 12 treatments were mainly manifested during the period of shelf life. Thus, the evaluation of the performances indicated by edible fruit rate (EFR) and marketability rate (MR), which were checked once a day during a 5‐day shelf. The fruits with no moldy and rotten appearance but completely browned pericarp were considered as edible fruits, and the fruits with no moldy, rotten, and browning appearance were considered as commodity fruits. During the shelf period, the MR and EFR of each treatment were the same, but the MR of CK‐48 hr (CK after 48‐hr storage) was 0 from the second day because of its severe pericarp browning.

(1) $$\begin{array}{c} \text{ERF}\;(\%)=\;\text{number of no moldy and rotten fruits/total fruit number}\\ \times 100\% \end{array}$$

(2) MR(%)=number of comadity fruit/total fruit number×100%

### 2.8 Determination of content of total soluble solids (TSS %)

The TSS contents (%, w/w) of fruits after a 48‐hr storage within foam box at 25℃ and the fruits after a 4‐day shelf were determined. The aril of 30 fruits from each sample was used for juicing and determination of TSS by a Brix refractometer (PR‐32α; ATAGO Co.). Each analysis was subjected to three repeats.

### 2.9 Color measurement

According to our previously reported method (Han et al., 2015), the chroma parameters including L*, a*, b*, C*, and h° of fruits after a 48‐hr storage within foam box at 25℃ and the fruits after a 4‐day shelf were determined using an automatic color analyzer (KONICA MINOLTA CR‐300). L* value represents lightness (+) to darkness (−), a* value represents red (+) to green (−), and b* value represents yellowness (+) to blueness (−), and the h° and C* values mean hue angle and saturation. Fifteen fruits from each treatment were measured. All treatments were performed in three biological repeats.

### 2.10 Measurement of contents of chlorophyll a, chlorophyll b, and carotenoid

According to the method of Ye and Chen (2004), 1.0 g frozen pericarp powder was weighed and mixed with 2 ml extracting solution (ethanol: acetone: water = 4.5:4.5:1.0) (V:V:V). The sample was ground, and the upper solution was transferred and filtered into a 25‐ml tube. The residue was ground again and washed for four times with total 8 ml extracting solution, and the upper solution was collected and filtered into a 25‐ml tube. The volume of collected filtrate was set to 10 ml with extracting solution. Then, the extract was used for determining the absorbance values at 663, 646, and 470 nm, respectively. The concentrations of chlorophyll a (Chl a), chlorophyll b (Chl b), and carotenoid (Car) in pericarp were calculated according to equations (3), (4), (5):

(3) $$\text{Chl}\;a\;\text{content}\;\left(\mu /g\right)\;=\;\left(12.21\;\times A_{663\;nm}\;-\;2.81\times \;A_{646nm}\right)\;\times \;10$$

(4) $$\text{Chl}\;b\;\text{content}\;\left(\mu /g\right)\;=\;\left(20.13\;\;\times \;A_{646\;nm}-5.03\;\times \;A_{663\;nm}\right)\;\times 10$$

(5) $$\begin{array}{c} \text{Carcontent}\;\left(\mu /g\right)\;=\;\left(10^{3}\times A_{470\;nm}-3.27\times \text{Chl}\;a-104\times \text{Chl}\;b\right)/229\;\\ \times \;10 \end{array}$$

### 2.11 Measurement of flavonoid content

One gram of frozen pericarp was ground, extracted, and washed by 80% (v/v) methanol. The extract was filtered, and its volume was set to 10 ml by 80% methanol. The content of flavonoid (Fla) was measured by the differential spectrophotometry method (Kang, 2005). 2% (w/v) zirconium dichloride (ZrOCl_2_.8H_2_O) in methanol solution was used as the complexant of flavonoid. 1.0 ml extract was transferred into a 15‐ml tube and then fully mixed with 7.0 ml methanol (solution A). 1.0 ml 2% ZrOCl_2_.8 H_2_O and 6.0 ml methanol were added into another 15‐ml tube containing 1.0 ml extract and fully mixed (solution B). After a standing for 1 hr, the absorbance at 420 nm of solution A (A_420 nm_ A) and solution B (A_420 nm_ B) was measured by a UV–Vis spectrophotometer (Mapada UV‐1800). A calibration curve was prepared for total flavonoid using rutin standard solution (52 µg/ml). A regression equation was obtained with a linear correlation coefficient *r* = .9999 and a linear range within 3.25 to 19.50 µg/ml. The total flavonoid content was calculated according to equation (6):

(6) $$\begin{array}{c} \text{Total}\;\text{flavonoid}\;\text{content}\;\left(\mu /g\right)=\left(A_{420nm}B-A_{420\;nm}A-\;0.0051\right)/\;0.0065\\ \times 10 \end{array}$$

### 2.12 Determination of sulfite residue

The sulfite residue (content, expressed as SO_3_^2‐^) was measured using a previously reported method (Luo, Niu, et al., 2019). 1.0 g pericarp or 5.0 g aril was ground into powder with liquid nitrogen and extracted using 2.0 ml saturated ZnSO_4_ solution and 5.0 ml saturated Na_2_B_4_O_7_.10H_2_O solution. The filtrate of the sample was transferred into a 100‐ml volumetric flask. Then, 4.0 ml 0.5 M NaOH solution was added and shaken. After a reaction for 5 min, 4.0 ml acid solution (H_2_SO_4_: deionized water = 1:71) was added and shaken. After a reaction for 2 min, 20 ml tetrachloromercurate sodium solution (absorbent, comprised of 1.36% (w/v) HgCl and 0.6% (w/v) NaCl) was added and fully shaken, and then, the volume was made up to 100 ml by deionized water. After be filtrated, the solution was used for the determination of sulfite residue. 5.0 ml tetrachloromercurate sodium solution, 1.0 ml formaldehyde (2.0 g/L), and 1.0 ml pararosaniline solution (0.02%, w/v, pararosaniline hydrochloride in 7.2% [v/v] HCl) were fully mixed with 2.0 ml sample solution and stood still for 20 min. The absorbance of solution at 550 nm was measured by a spectrophotometer (Mapada UV‐1800). A calibration curve was prepared with SO_2_ standard solution (2 µg/L) made from NaHSO_3_. A regression equation was obtained with a linear correlation coefficient *r* = .9996 and a linear range within 0.11–0.78 µg/ml. The sulfite residue was calculated based on equations (7) and (8):

(7) $$\begin{array}{c} {\text{SO}}_{2}\;\text{in}\;\text{pericarp}\;\left({\text{SO}}_{2}-P,\;\mu g/g\;\text{FW}\right)=\left(A_{550\;nm}-\;0.0035\right)\;\\ \times \;100/\;\left(0.0356\;\times \;2\;\times \;1\right) \end{array}$$

(8) $$\begin{array}{c} {\text{SO}}_{2}\;\text{in}\;\text{aril}\;\left({\text{SO}}_{2}-A,\;\mu g/g\;\text{FW}\right)=\left(A_{550\;nm}\;-0.0035\right)\\ \;\times \;100/\;\left(0.0356\;\times \;2\;\times \;5\right) \end{array}$$

### 2.13 Statistical analysis

Compared with the harvest day of CK (marked as CK‐0 day), changes in chroma values, TSS%, pigment contents after 48‐hr storage, and 4‐day shelf were all expressed as the differences (Δ) between the measured day and CK‐0 day, that was, Δvalue = measured value ‐ average value of CK‐0 day. Among them, the “STΔ chroma value” meant “after 48‐hr storage,” “SFΔchroma value” meant “after 4‐day shelf.” The excel software was used to calculate the Δvalue's standard errors (SE), which are equal to those of measured values.

Multiple comparisons were performed using Duncan's new complex‐difference method in SPSS 19.0. To comprehensively and intuitively assess the fruit storability, the factor analysis method in SPSS 19.0 was used to not only reduce the dimensionality of EFR and MR data, but also evaluate the overall quality of fruits after a 48‐hr storage (OQST) and the fruits after a 4‐d shelf (OQSF). Based on the common factors and their contributions calculated by variables (the EFR and MR data of 3–5‐day shelf), the evaluation scores of EFR (or MR) for all of 12 treatments and CK‐48 hr were obtained by ∑(common factor × contribution). In the same way, the evaluation scores of OQST were calculated by Δchroma values and ΔTSS after 48‐hr storage; those of OQSF were calculated by EFR score, Δchroma value, and ΔTSS after 4‐day shelf.

The orthogonal experiment data were analyzed by the general linear model in SPSS 19.0, and the descriptive statistics and multifactorial multivariate analysis of variance were performed on the trial results.

## 3 RESULTS

### 3.1 Changes in TSS contents and chroma values in longan fruit after a 48‐hr RT storage

**Figure 1:**
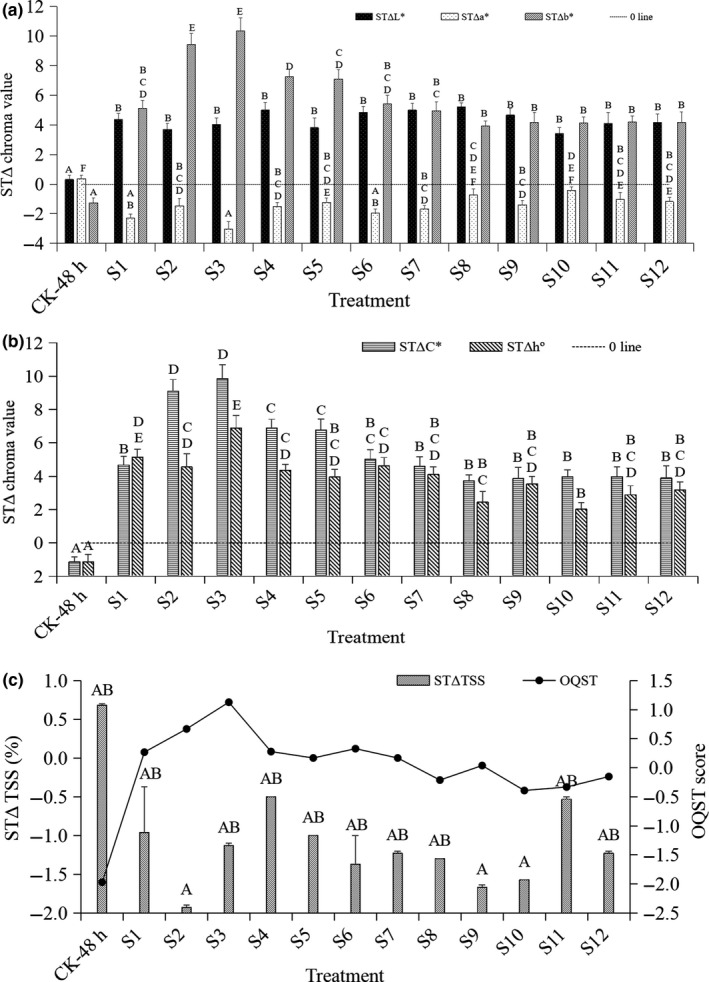
Changes in chroma values (a–b), TSS & OQST score (c) of the control and treated longan fruits after a 48‐hr storage within foam boxes at 25℃. Note: The superscript letters on the columns marked with same pattern indicated significant differences between the treatments and CK‐48 hr at α = 0.01 (*p* < .01), and the following was the same. *Note*: The TSS % and chroma values of CK‐0 day were used as the reference values: L*: 51.04 ± 0.08 A; a*: 6.54 ± 0.06 EF; b*: 31.50 ± 0.11 A; C*: 32.19 ± 0.10 A; hº: 78.24 ± 0.15 A, which were represented with 0 line in the figures, and the following was the same

As shown in Figure 1, after a 48‐hr storage, the TSS % showed no difference between CK‐48 hr and CK‐0 day (*p* > .01), and the TSS % of S1, S3, S4, S5, S6, S7, S8, S11, and S12 was decreased with no significant difference (*p* > .01) (Figure 1c). The L^*^, b^*^, C^*^, and h^°^ values of S1 to S12 were significantly higher than those of CK‐48 hr and CK‐0 day (*p* < .01), but the a* values of most of the treatments were significantly lower than those of CK‐48 hr and CK‐0 day (*p* < .01) except for S8 and S10 (*p* > .01) (Figure 1a‐b). Thus, the deeper greenish‐brown pericarp of CK‐48‐hr fruits compared with the pericarp of CK‐0 day fruits might be resulted from the lower L*, b*, C*, and h° values and higher a* value (*p* > .01). More importantly, compared to CK‐48‐hr fruits, better appearance quality (bright yellowish‐green pericarp) of the fruits from 12 treatment groups was consistent with their higher overall quality scores after a 48‐hr storage (OQST) (Figure 1c).

### 3.2 Changes in pigment contents in pericarp of longan fruit after a 48‐hr RT storage

**Figure 2:**
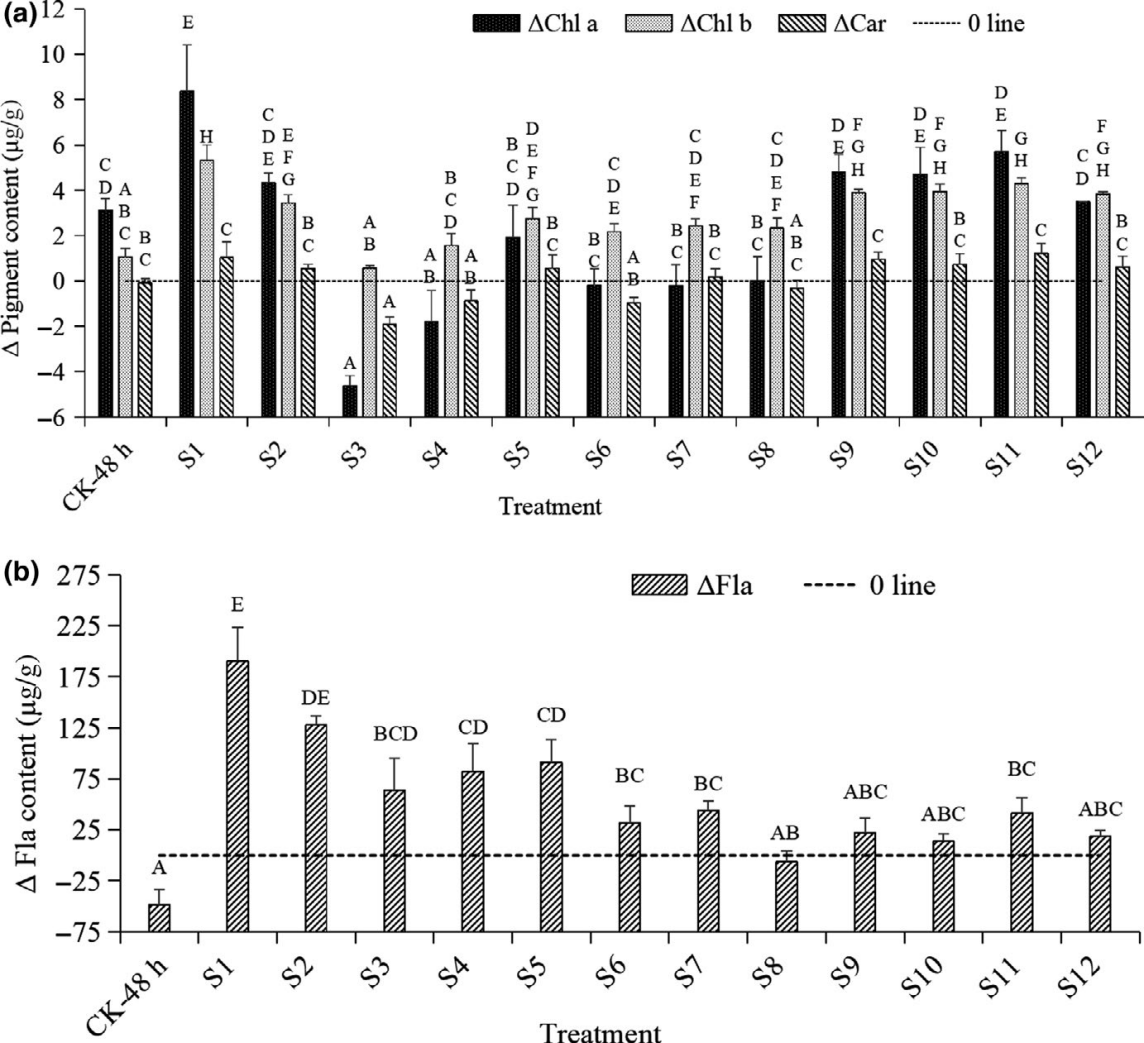
Changes in contents of pigment (a) and flavonoid (b) of the control and treated longan pericarp after a 48‐hr storage within foam box at 25℃. *Note*: The pigment contents in longan fruit pericarp of CK‐0 day were used as the reference value (mg/g): Chl a: 39.81 ± 0.21 BC; Chl b: 12.11 ± 0.05 A; Car: 13.41 ± 0.19 BC; Fla: 253.73 ± 4.69 AB

As shown in Figure 2a, compared with those in CK‐0‐d pericarp, except the Chl a content of S3 decreased significantly (*p* < .01), S1 and S9–S11 increased significantly (*p* < .01), the others had no significant differences (*p* > .01). The contents of Chl b were all increased, and most of them were significantly higher than those of CK‐0 day and CK‐48 hr (*p* < .01). But the Car content showed no significant difference except for S3. Besides, CK‐48 hr showed higher chl a and chl b contents than CK‐0 day (*p* > .01). In Figure 2b, the Fla contents in CK‐48‐hr pericarp decreased with no significance (*p* > .01). But the Fla contents of 4 treatments (S1, S2, S4, and S5) were significantly higher than that of CK‐0 day (*p* < .01). The above results indicated that most of the treatments (especially S1) helped maintain chlorophyll (mainly Chl b) and Fla contents in the longan pericarp during a 48‐hr storage in foam box at 25℃.

### 3.3 SO_2_ residues in longan fruit after a 48‐hr RT storage

**Figure 3:**
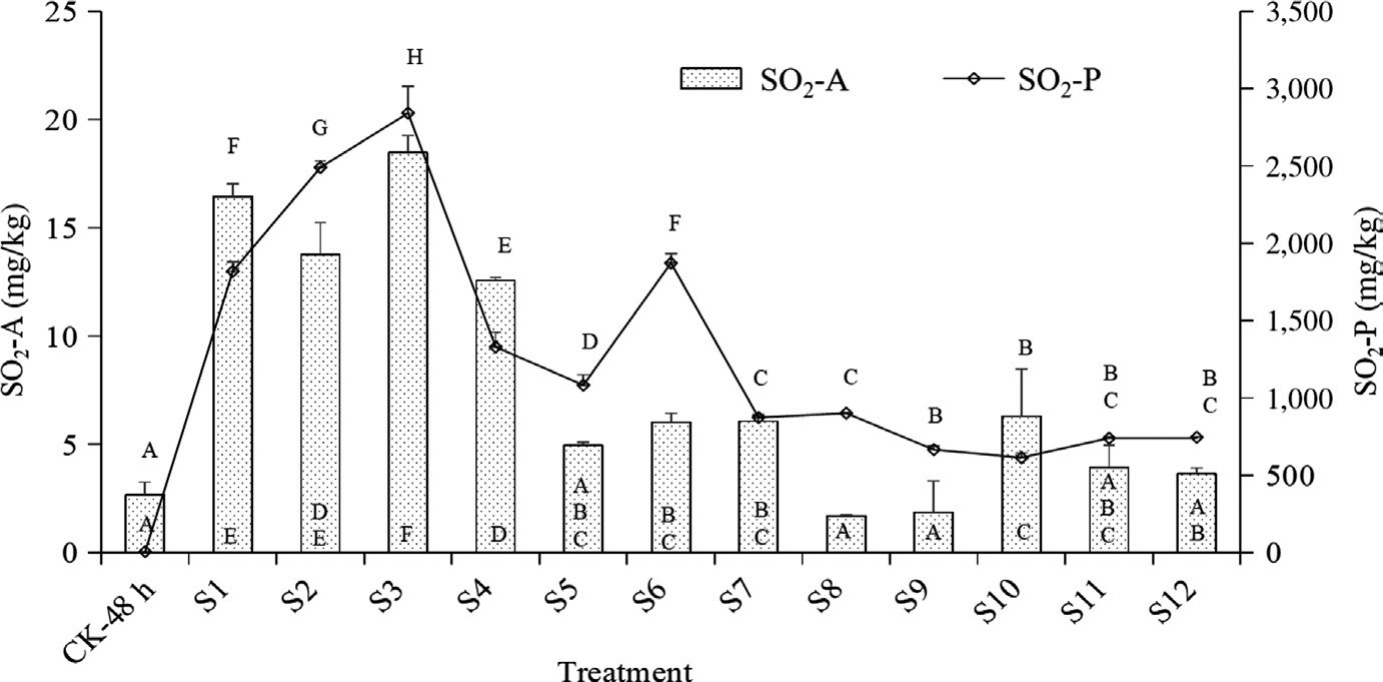
SO_2_ residues of the control and treated longan fruits after a 48‐hr storage within foam boxes at 25℃. *Note*: The superscript letters indicated significant differences in SO_2_‐P at α = 0.01 (*p* < .01), and the subscript letters were set for SO_2_‐A

The SO_2_ residues in the pericarp (SO_2_‐P) of treated longan fruits ranged from 615.23 ± 30.96 to 2,841.81 ± 173.62 mg Kg^‐1^, but the SO_2_ residues in the pulp (SO_2_‐A) ranged from 1.73 ± 0.05 to 18.47 ± 0.79 mg Kg^‐1^. Significant differences were also observed between the treatments and CK‐48 hr (*p* < .01) (Figure 3). These results indicated that SO_2_ residue was mainly accumulated in pericarp and little in aril during the 48‐hr storage, and the SO_2_‐A residues of treatments were all below the national limit (≦30 mg Kg^‐1^).

### 3.4 Performance and evaluation of the apparent and internal quality in longan fruits during the shelf life

**Figure 4:**
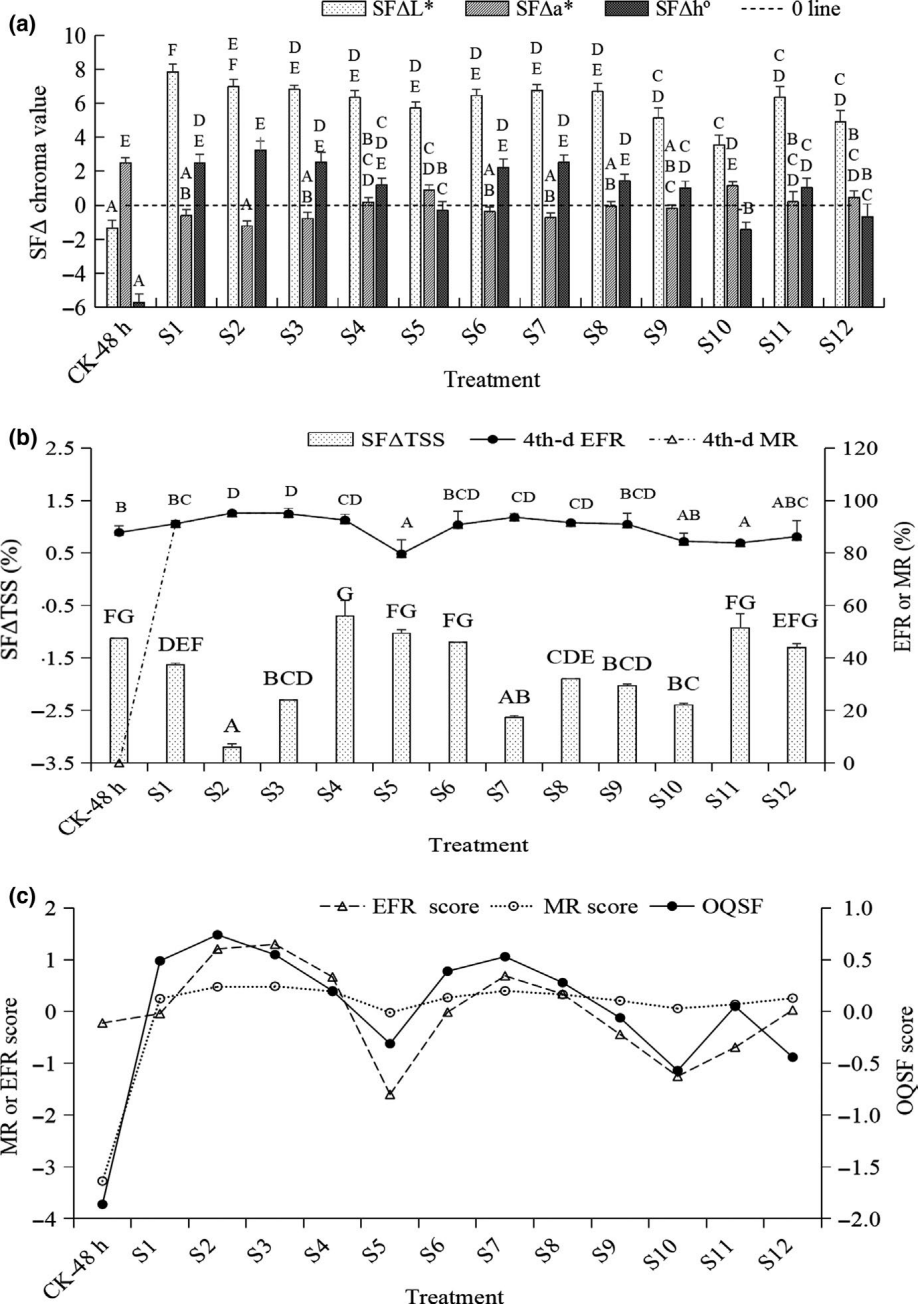
Changes in chroma values (a), TSS%, edible fruit rate (EFR, 4th‐day), and marketability rate (MR, 4th‐day) (b); and the evaluation scores of EFR (3–5 day), MR (3–5 day), and the overall quality scores (OQSF) (c) of the control and treatments after a 4‐day shelf. *Note*: The TSS% of CK‐0 day was used as the reference value: 22.3 ± 0.06 H; the 4th‐day EFR of CK‐48 hr was 87.82%, but its 4th‐day MR was 0; the 4th‐day EFR and 4th‐day MR of S1–S12 were the same, respectively

As shown in Figure 4a, compared to CK‐0 day, CK‐48 hr showed a significant decrease in L^*^ and h^º^ values but increase in a^*^ value after a 4‐day shelf. All of the 12 treatments showed higher L* values than CK‐0 day and CK‐48 hr, and lower a* values and higher h^º^ were observed in S1, S2, S3, S6, and S7. The above results explained that the treated fruits after a 4‐day shelf life still maintained a better exterior quality than CK‐48 hr and CK‐0 day. Figure 4b showed that TSS% in fruits of CK‐48 hr and 12 treatments was significantly lower than that in fruits of CK‐0 day (*p* < .01). Moreover, TSS% in fruits of S2, S3, S7, S8, S9, and S10 (but not other 6 treatments) was significantly lower than that in CK‐48 hr (*p* < .01). Except for S5 and S6, the 4th‐day EFRs (MRs) of S2–S9 were all higher than that of CK‐48 hr (*p* < .01). Because CK‐48‐hr pericarp was severely browned, its 4th‐day MR was 0 without commercial value any more. Among S1–S12, except for S2, S3, S5, and S11, there was no significant difference in 4th‐day MR (*p* > .01).

In Figure 4c, CK‐48 hr had a higher EFR score than 4 of 12 treatments, but it had much lower MR than 12 treatments. So, it could be concluded that the storability difference between CK‐48 hr and 12 treatments was mainly due to the apparent quality, CK‐48 hr had a much lower OQSF score than 12 treatments because of its poor appearance, that is, all of its fruits were completely browned.

### 3.5 Results of multivariate test for 3‐test factors in the orthogonal experiment

Using all the detected indicators as the dependent variables and using chroma values as dominant ones, four‐test methods including Pillai's track, Wilks's λ, Hotelling's track, and Roy's maximum root were performed on the 3‐test factors. Because Roy was the largest eigenvalue in the Hotelling test matrix, the significance values (*p* value) of the two tests were the same.

As shown in Table 3, the results gave the effect of each factor on the whole trial, the *p* values of the four tests for the three factors were all in the order of A < C < B. This suggested that FacA had the greatest effect on the storability difference between 12 treatments, reaching a significant level (*p* < .05 in 2‐test methods), then followed by FacC (*p* = .05–.10 in two tests), and FacB had the least effect (*p* > .10).

**Table 3:** Multivariate test for the significance of three factors based on all the indicators (dependent variables) dominated by chroma values^c^

Effect	Value	*F*	Hypothetical *df*	Error *df*	Sig.
Intercept					
Pillai's track	0.999	473.497^a^	4	1	0.034
Wilks's λ	0.001	473.497^a^	4	1	0.034
Hotelling's track	1,893.987	473.497^a^	4	1	0.034
Roy's maximum root	1,893.987	473.497^a^	4	1	0.034
FacA (sodium metabisulfite)					
Pillai's track	2.46	3.42	12	9	0.037
Wilks's λ	0.001	3.309	12	2.937	0.181
Hotelling's track	−	−	12	−	−
Roy's maximum root	31.532	23.649^b^	4	3	0.013
FacB (precooling method)					
Pillai's track	1.672	2.55	8	4	0.191
Wilks's λ	0.024	1.354^a^	8	2	0.492
Hotelling's track	11.498	0	8	0	−
Roy's maximum root	7.843	3.921^b^	4	2	0.213
FacC (precooling duration) ((precooling duration)					
Pillai's track	1.793	4.332	8	4	0.087
Wilks's λ	0.008	2.555^a^	8	2	0.312
Hotelling's track	24.041	0	8	0	−
Roy's maximum root	18.631	9.315^b^	4	2	0.099

^a^ Accurate statistics.

^b^ This statistic was the upper limit of F, which produced a lower bound on the level of significance.

^c^ Design: intercept + FacA +FacB + FacC.

### 3.6 Results of main effect test based on all the detected indicators (dependent variables)

As listed in Table 4, the results from corrected model shown that the combined three factors had certain statistical significance for STΔb ^*^, STΔC ^*^, STΔh^º^, SO_2_‐P, and SO_2_‐A (*p* = .027–.097).

**Table 4:** Effective indicators (dependent variables) plus OQST and OQSF with significant or less significant differences, which were screened out from the main effect test

Source	Dependent variable	Type III sum of square	*df*	Mean square	*F*	Sig.
Corrected model	STΔb^*^	47.125	7	6.732	4.534	0.081
	STΔC^*^	44.471	7	6.353	4.408	0.085
	STΔh^º^	16.603	7	2.372	4.068	0.097
	SO_2_‐P	5,669,433.298	7	809,919.043	5.525	0.059
	SO_2_‐A	344.404	7	49.201	8.758	0.027
FacA	STΔb^*^	34.755	3	11.585	7.803	0.038
	STΔC^*^	31.961	3	10.654	7.391	0.041
	STΔh^º^	13.59	3	4.53	7.77	0.038
	SO_2_‐P	5,346,476.736	3	1,782,158.912	12.156	0.018
	SO_2_‐A	305.116	3	101.705	18.104	0.009
	OQST	2.553	3	0.851	2.727	0.178
	OQSF	2.973	3	0.991	3.128	0.150
FacB	OQST	0.322	2	0.161	0.517	0.632
	OQSF	0.038	2	0.019	0.061	0.942
FacC	STΔb^*^	10.890	2	5.445	3.667	0.125
	STΔC^*^	10.912	2	5.456	3.785	0.120
	OQST	0.058	2	0.029	0.093	0.913
	OQSF	0.626	2	0.313	0.988	0.448

For FacA, there were some significant differences between three levels of chroma values, Fla contents, and SO_2_ contents after 48‐hr storage (*p* = .009–.075), but there were no significant differences in OQST and OQSF (*p* = .178 and .150). For FacB, OQST (*p* = .632) had a more significant difference than OQSF (*p* = .942). For FacC, there was a less significant difference between three levels of STΔb^*^ and STΔC^*^, and the OQST difference (*p* = .913) was lower than OQSF (*p* = .448). For all of the three factors, the level differences in OQST and OQSF were not significant (*p* = .150–.942).

### 3.7 Average value comparisons of the effective indicators at different levels of each factor

Because OQST and OQSF are the primary indicators used for choosing the appropriate level of each factor, the relevant indicators to them were also screened out and analyzed on the basis of their average values of different levels of each factor (Table 5).

**Table 5:** Comparisons of average values of the effective indicators (plus OQST and OQSF) at different levels of three factors

	Level	SO_2_‐A	SO_2_‐P	STΔa^*^	STΔb^*^	STΔC^*^	STΔh^º^	ΔFla	OQST	OQSF
FacA	0.22%	16.22	2,384.13	−2.27	8.29	7.87	5.53	127.41	0.59	0.63
	0.18%	7.85	1,428.91	−1.57	6.58	6.23	4.31	68.42	0.27	0.04
	0.14%	3.22	814.41	−1.27	4.34	4.06	3.36	19.74	−0.24	0.09
	0.12%	4.64	701.22	−0.88	4.16	3.94	2.70	24.41	−0.62	−0.76

	Level	STΔL^*^	STΔa^*^	STΔb^*^	STΔh^º^	ΔFla	SO_2_‐P	SO_2_‐A	OQST	OQSF
FacB	UP	4.54	−1.45	5.11	3.83	85.85	1,142.58	10.25	−0.02	0.07
	PP	4.13	−1.09	6.49	3.48	62.55	1,302.24	6.22	−0.20	0.01
	WP	4.43	−1.95	5.93	4.62	31.59	1,551.69	7.47	0.22	−0.08

	Level	SFΔTSS	STΔb^*^	STΔC^*^	ΔFla	SFΔL^*^	SFΔa^*^	SFΔh^º^	OQST	OQSF
FacC	4 hr	−1.39	4.49	4.17	63.68	6.81	−0.23	1.81	0.02	0.32
	12 hr	−1.83	6.23	5.93	69.72	5.91	−0.15	1.18	−0.10	−0.09
	20 hr	−2.09	6.81	6.48	46.58	5.69	0.12	0.82	0.07	−0.23

Note

The additional effective indicators for FacB and FacC were selected because of their certain effects on the OQST and OQSF, respectively.

Firstly, the higher the level of FacA, the higher the STΔb^*^, STΔC^*^, STΔh^º^, and ΔFla; the lower the STΔa^*^, the better the OQST and OQSF; and the higher the SO_2_ residues in fruit, but those in aril were all under the national standard limit (30 mg/kg). So, A_1_ was selected.

Secondly, FacB mainly affected the OQST. Among three precooling methods, UP (uncovered precooling) gave the highest STΔL^*^, ΔFla, and SO_2_‐A; lower STΔb^*^ and STΔC^*^; medium STΔh^º^ and OQST, but the best OQSF. So, B_1_ was selected.

Finally, FacC mainly affected the pigment contents and OQSF. With the prolonging of precooling duration, although STΔb^*^ and STΔC^*^ showed an upward trend, ΔFla, SFΔL^*^, and SFΔh^º^ were all the opposite, and SFΔa^*^ raised, this led to a worse fruit appearance. Meanwhile, the SFΔTSS% declined, and OQSF also dropped. So, C_1_ was selected. Therefore, the reports in the above suggested that A_1_B_1_C_1_ (0.22% compound preservative + uncovered precooling + 4 hr precooling) was a more reasonable treatment combination.

## 4 DISCUSSION

During the storage at room temperature (25℃), the higher temperature of longan fruits can cause a lot of breathing heat let out which will be accumulated inside the package little by little, and more and more condensated water will gather on the surface of fruit, which can easily lead to the browning of pericarp after the fruit is taken out and dried. So, a timely precooling after harvest is essential to cool the fruits and remove the respiratory heat inside them, and reduce the dewing on the internal surface of package (Brosnan & Sun, 2001; Wang, Chen, Yu, & Ji, 2014). Although the treatments by environmentally friendly compounds such as microencapsulated essential oils (Alikhani & Daraei Garmakhany, 2012) were encouraged and the residue from SO_2_ treatment might be harmful to human health, the sulfur dioxide fumigation was proved to be the most effective preservation method for longan in the export trade. Slow and continual release of SO_2_ was an important improvement to reduce sulfur residue in the recent years. In this work, SO_2_‐released paper was used and combined with low‐temperature precooling to enhance longan fruit storability which were stored in foam boxes at 25℃ room temperature, and the desired effects were achieved as shown by not only drastically improving the OQST but also maintaining an excellent OQSF during 5‐day shelf period. In spite of the fact that UP could lead to a more water loss and a darker appearance, the cooling was quicker and beneficial to protect the OQST and OQSF. Maybe NP and WP give a better chroma quality after 48‐hr storage, but resulted in a worse OQSF, as a longer‐term precooling would do. And the longer the precooling duration, the lower the TSS content in aril. So, the comprehensive analysis showed that the combination of A_1_B_1_C_1_, that is, “0.22% compound preservative + uncovered precooling + 4 hr precooling” was a more appropriate treatment and worthy of further optimization research.

The preservation effect of SO_2_ on longan fruits lies in the protection of appearance and internal quality. Its mechanism of action includes the viewpoints of antioxidation and antibrowning (Wu et al., 1999; Pang, Zhang, Gong, & Zhang, 2007; Han, Wu, Ji, & Han, 1999), and delaying the TSS content decline to some extent (Pang, Zhang, Gong, & Zhang, 2007). However, the changes in the color‐related pigment contents have not been understood in details before and after treatment with SO_2_. It has been reported that appropriate SO_2_ treatment could facilitate the accumulation of anthocyanins (Liu, Wang, Yi, & Yi, 2015), polyphenols, flavonoids, and lignin in *Vitis vinifera* during storage (Xue & Yi, 2017), but promoted the degradation of flavonoid glycosides in dried chrysanthemum morifolium cv. Hangju (Wang, Hao, et al., 2014), whereas the antioxidant activity of flavonoids before oxidation was higher than that after oxidation (Li, Long, Li, & Hao, 2012). As described in this paper, although all the fruits of 12 treatments had a significantly higher RECP than the control, their pericarp browning was instead inhibited. The Fla contents in the pericarp were significantly increased, even higher than CK‐0 day. At the same time, the STΔa^*^ decreased, but the STΔb^*^, STΔc^*^, STΔh^°^, and SFΔL^*^ all went up. Apart from the bleaching of pigments such as anthocyanins, it was presumed to be related to the reduction in oxidized flavonoid or polyphenols by SO_2,_ suitable low‐temperature precooling also protected the pigments such as total chlorophyll and flavonoid from rapid degradation, and reduced the formation of carotenoid, so that the process of oxidative browning of longan fruit pericarp was delayed. In addition, the TSS content had no significant relationship with the chroma quality and MR, which indicated that the relationship between the TSS content and longan fruit storability is not notable.

In brief, among three factors, FacA is the most important to the OQST and OQSF of longan fruit, and FacB and FacC play an auxiliary role; FacB mainly affects the OQST, and FacC affects the OQSF. What's more, the follow‐up observations showed that the longest shelf life among the treatments reached 10 day, so the 4‐day shelf life could not adequately demonstrate the differences between 12 treatments in OQST and OQSF, which explained the results of OQST and OQSF with no marked difference in this study, and provided a future reference for predicting the fruit shelf in the similar trials.

## 5 CONCLUSION

From this study, it could be concluded that SO_2_‐released paper combined with low‐temperature precooling was able to significantly improve the OQST and OQSF of longan fruits after 48‐hr storage within foam box at 25℃, featured with a shelf life of more than 4 days. The combined treatment “0.22% compound preservative + uncovered precooling + 4 hr precooling” was a suitable treatment, which helped to increase the total chlorophyll and Fla content significantly, and consequently improve the chroma levels after 48‐hr storage and during the shelf, but the changes in TSS content and RECP had no significant correlations with them. The results of this work would contribute to developing a new application of SO_2_ in longan fruit preservation technology and making more progress in the postharvest technology of longan fruit.

## CONFLICT OF INTEREST

The authors declare that they have no conflicts of interest.

## ETHICAL APPROVAL

This study does not involve any human or animal testing.
